# Social media and partnership jointly alleviate caregivers’ psychological distress: exploring the effects of online and offline connectedness

**DOI:** 10.1186/s40359-023-01415-9

**Published:** 2023-11-14

**Authors:** Song Harris Ao, Luxi Zhang, Piper Liping Liu, Xinshu Zhao

**Affiliations:** 1 Department of Communication / Institute of Collaborative Innovation / Center for Research in Greater Bay Area, University of Macau, Macau, China; 2 Department of Communication / Institute of Collaborative Innovation, University of Macau, Macau, China

**Keywords:** Caregiving, Psychological distress, Alcohol drinking, Social media, Partnership, Percentage coefficient (*b*_*p*_), Mediation analysis, Moderated moderation analysis

## Abstract

**Background:**

The prevalence of caregiving in the United States has increased from 16.6% to 19.2% during the period between 2015 and 2020. Caregivers play a critical public health role post-pandemic and as the population ages. However, caregiving can be detrimental to the health of caregivers. Many studies have shown that communication and connectedness are effective forms of health intervention for caregivers, but how this can be achieved requires further investigation.

**Objective:**

This study aimed to investigate the indirect effect of caregiving on problems of alcohol drinking through psychological distress. Moreover, this study aimed to provide initial evidence of the distinct effects of online and offline communication and connectedness on caregivers' well-being.

**Methods:**

The predictions were evaluated by examining responses to the Health Information National Trends Survey 2020 (*n* = 3,865). A mediation analysis was conducted to test the mediating effect of psychological distress on the association between caregiving and alcohol drinking. A second-level moderation analysis was performed. The online communication and connectedness, social media use for health, and the offline type, marital or romantic partnership, were tested as moderators to lessen the psychological distress of caregiving.

**Results:**

A competitive mediation was identified. We found a positive indirect effect from caregiving to alcohol drinking mediated by psychological distress (*b*_*p*_ = .0017, *p* < .05) but a negative direct effect from caregiving to alcohol drinking (*b*_*p*_ = -.0340, *p* < .05). Furthermore, the study reported a strongly positive effect of moderated moderation on the linkage from caregiving to psychological distress. The negative impact of caregiving on mental distress was greater among those who used social media less, particularly those without a romantic or marital partner.

**Conclusions:**

The findings indicate that caregivers experience more mental distress, which leads to risky behavior. This study highlights the crucial role of both online and offline connectedness in mitigating the adverse consequences of caregiving.

## Background

The American population aged 65 and over increased by 15.5 million from 2010 to 2020, as indicated in the 2020 Census [1]. This surge has heightened the caregiving burden. According to the 2020 Caregiving Report conducted by the National Alliance for Caregiving (NAC), the percentage of adults identified as caregivers increased from 16.6% in 2015 to 19.2% in 2020, with more than one in five adults being caregivers [2]. Caregivers provide essential support to the social health system. However, caregivers’ heavy workloads often lead to psychological distress, encompassing depression and anxiety [3, 4]. The COVID-19 pandemic has put extra pressure on caregiving [5]. As NAC addressed in its caregiving report in 2020, the self-evaluated health condition of caregivers has deteriorated from 2015 to 2020. In 2017, 17% of caregivers reported their health status as fair or poor, compared with 21% in 2020 [2]. No such deterioration was observed in the overall U.S. population [2, 6].

Psychological distress might influence behavioral health [7]. For instance, sometimes caregivers tend to increase alcohol consumption to cope with mental distress [4, 8, 9]. While previous research has predominantly focused on the health status of care recipients [7, 10], there has been limited focus on addressing the mental and behavioral health risks that caregivers encounter. We aim to fill this gap by clarifying how caregiving leads to alcohol consumption, mediated through its impact on psychological distress. Furthermore, we aim to investigate practical measures that can enhance caregivers’ well-being. Communication and connectedness have been identified as beneficial for caregivers’ mental health [11, 12], potentially acting as moderators to mitigate the adverse effects of caregiving burden on caregivers’ mental and psychical well-being [13, 14]. In the digital era, both online and offline forms of communication and connectedness are available to caregivers [13, 14]. Social media use for health refers to individuals' behaviors of generating and sharing health-related content, seeking health support on social media platforms [15]. Therefore, the specific use of social media for health, representing the online dimension of connectedness, offers caregivers a platform to engage with peers in similar caregiving roles and receive support [13]. The marital or romantic partnership, representing offline connectedness, provides caregivers with intimate companionship and support [16, 17]. However, more in-depth exploration of how these different forms affect the mental health of caregivers still needs further investigation. Our study stands as one of the first to probe into such effects and propose practical measures for improving caregivers’ mental health.

This study has two primary objectives. First, we address the mental and behavioral health risks associated with caregiving, with a specific focus on the impact of caregiving on alcohol drinking. Psychological distress serves as a mediator to explain the underlying mechanism of the association between caregiving and alcohol drinking. Second, by incorporating online and offline connectedness as moderators to alleviate caregivers’ mental distress, we explore health interventions aimed at enhancing caregivers’ well-being.

### Health outcomes of caregiving: mapping caregiving, psychological distress and alcohol drinking into a mediation model

Caregiving refers to the provision of care or healthcare decisions made for others, including patients, family members, friends, and non-relatives [18, 19]. In some cases, caregivers provide multiple types of care: for example, an adult caregiver may provide care to both children and older family members [18, 20]. Those caregivers who provide care to more than one recipient are defined as compound caregivers [21, 22]. In 2020, adult compound caregivers reached 24% in the U.S., with a 6% increase compared with 2015 [2]. The additional caregiving responsibilities undertaken by compound caregivers contribute to heightened caregiving burdens [13, 14]. In recent years, there has been growing concern about the negative health outcomes of caregiving, especially for those facing substantial caregiving burdens [3, 23, 24].

Studies have shown that caregiving can trigger mental health problems [3, 4], due to the heavy physical and mental workload [19]. The responsibilities of caregivers impose an additional resource burden, in terms of time and energy [25, 26]. Empirical studies have shown that caregivers often experience psychological distress, including depression and anxiety [27, 28], and even mental health disorders [13, 29]. In addition, compound caregivers reported more mental distress than non-compound caregivers [30]. We therefore propose our first hypothesis:

- H1. Caregiving is positively associated with psychological distress. (*a* path).

Psychological distress is often associated with risky behavior [4, 8, 9], as such behavior acts as an avoidance mechanism to cope with depressed and anxious emotions [8, 31]. Some caregivers may increase their level of alcohol drinking to numb their emotional pain and alleviate mental distress. Alcohol consumption has been identified as a result of the psychological distress caused by caregiving [8, 18, 31–33], indicating a mediation effect of psychological distress. Thus, we propose our next two hypotheses:

- H2. Psychological distress is positively associated with alcohol drinking. (*b* path).
- H3. Caregiving is positively associated with alcohol drinking, as mediated by psychological distress. (a*b path).

A “direct path”, or *d* path, may also be involved in the mediation model. However, the *d* coefficient estimates not only the strictly defined direct effect but also all indirect effects not captured by mediation through psychological distress. The term “direct and remainder path” would thus be more accurate [34–36]. Some studies have shown that caregiving can lead to poor sleep [37], which can lead to an increase in alcohol consumption [38], indicating a positive indirect link between caregiving and alcohol drinking via insomnia. Other studies, however, have indicated that caregiving tasks reduce personal free time, which leads to less alcohol drinking, thus indicating a negative indirect effect of caregiving on alcohol drinking [18, 33]. There are indeed mediators other than psychological distress that can explain the relationship between caregiving and alcohol drinking, but limited knowledge exists regarding the combined effect of numerous mediators when controlling for the effect of psychological distress. We therefore consider this path as an inter-hypothesis research question.

Q1. What is the relationship between caregiving and alcohol drinking when controlling the effect of psychological distress? Positive, negative, or statistically inconclusive? (*d* path).

Without controlling for the effect of psychological distress, the relationship between caregiving and alcohol drinking is the total effect under examination. Findings of prior studies about whether caregiving can affect alcohol drinking are not consistent [4, 8, 18, 20]. Some studies have pointed out that caregiving can increase problematic alcohol drinking, as caregivers may turn to alcohol to escape from their reality and ease their burden [18, 39]. For example, a study investigating spousal caregivers showed that 34.1% reported alcohol use as a way to cope with the stress derived from providing care [32]. Other studies have reported a negative or statistically inconclusive association between caregiving and alcohol drinking [4, 8], and that the time taken up by caregiving probably reduces caregivers’ free time to drink or take part in social activities [18, 33]. Thus, we propose our second inter-hypothesis research question.

Q2. What is the relationship between caregiving and drinking without controlling for psychological distress? Positive, negative, or statistically inconclusive? (*c* path).

### Communication and connectedness as health interventions: exploring the moderating effects

Current findings regarding public health have revealed that connectedness and communication are effective tools for protecting health [16, 40]. They can slow down the release of chemicals that affect the immune system, such as cortisol and cytokines [12]. Connecting and communicating help to prevent the overactivation of the hypothalamic–pituitary–adrenal axis [11, 12, 41], and can provide caregivers with emotional support and opportunities to express negative feelings [11, 12]. The diversity of social communication and connectedness is more beneficial than relying on single ties [42, 43]. Digital technology provides caregivers with more options to communicate, but the effects of specific means of communication and connectedness on caregivers have not been thoroughly examined. We examine this issue from the perspectives of social media as the online form and marital or romantic partnership as the offline form.

Over the past two decades, social media has emerged as a method of providing interventions in public health [44] and facilitating social connections and communication [45]. The social media environment enables people to communicate freely, develop and maintain relationships, and achieve a sense of belonging [46, 47]. During the COVID-19 pandemic, social media was considered a primary source of health information for those facing social isolation [48]. A negative link between social media use and caregiving burden has been found [13, 49, 50], with caregivers who participate in a social health forum reporting less burden of caregiving and psychological distress than those who only use the Internet, as they can communicate with peers and obtain support through social networks [13]. Thus, social media use for health, which represents an online form of communication and connectedness, can moderate the positive association between caregiving and psychological distress. Therefore, we propose our next hypothesis:

H4. Social media use for health negatively moderates the relationship between caregiving and psychological distress, i.e., higher social media use predicts a weaker positive association between caregiving and psychological distress.

Marital or romantic partnerships also serve as a means to enhance connectedness and communication. Romantic relationships can provide individuals support, companionship, and opportunities for interpersonal communication [16, 17]. Studies have shown that intimate connectedness is positively associated with a sense of meaning in life and mental health [51, 52].

Many scholars have examined the relationships between online and offline connectedness and communication [53, 54], but investigations into social media use and partnership relationships offer mixed results. Both a negative [55, 56] and a positive association [57, 58] have been found. Caregivers invest extensive time and energy in their care receivers [5, 24], so they have limited personal resources to engage in communication of any kind. The characteristics of online and offline communication are unique; for example, social media provides a flexible way for caregivers to communicate with various people when and where they want to, or help them to find caregiver communities and feel supported and connected [12, 13]. Caregivers with partners may benefit from direct company and interaction and enjoy intimacy and togetherness. Some studies have argued that offline communication and connectedness are of higher quality than online types, as they can provide physical interaction such as hugs and create a safer and more private form of intimacy [59]. Thus, caregivers with partners are likely to rely on them for emotional support rather than using social media, which will affect the moderation effect of social media use for health on the caregiving to psychological distress relationship. This informs our next hypothesis.

H5. Marital or romantic partnership positively moderates the social media use moderation effect, i.e., having a partner predicts less negative social media use moderation effects.

**Fig. 1:**
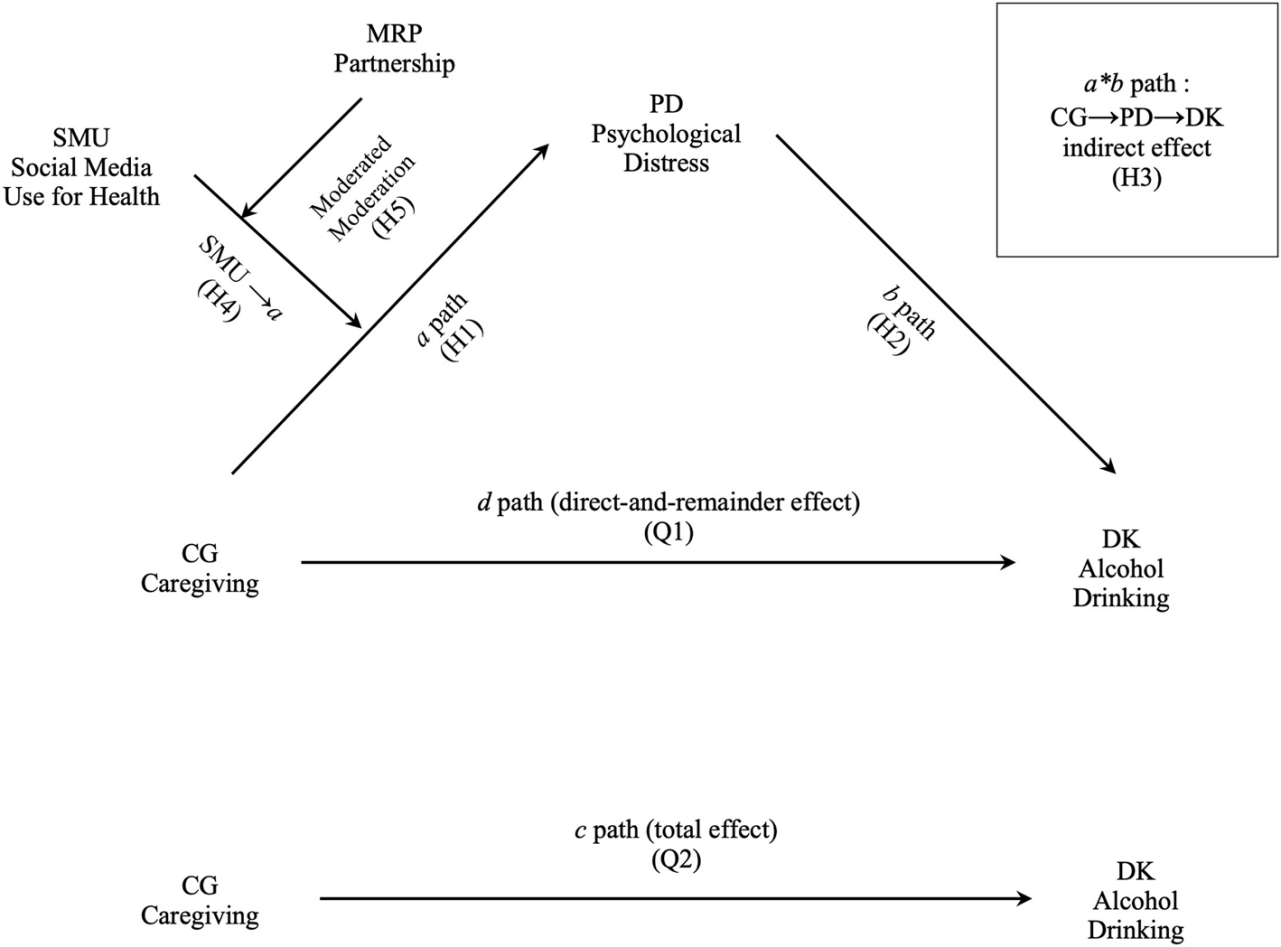
Conceptual model

See Fig. 1 for the conceptual model.

## Methods

### Data source and sample

The data were from the Health Information National Trends Survey collected between February and June 2020 (HINTS 5, Cycle 4, http://hints.cancer.gov/). HINTS is designed to acquire nationally representative data from U.S. adults, enabling the monitoring of behaviors of health communication and the development of efficacious strategies [60]. Employing a two-stage random sampling technique, HINTS achieved a response rate of 36.7% for the year 2020 [61]. A total of 3,865 participants responded to the postal-mail survey. Non-valid responses were deleted pairwise for regression analyses.

### Measures

The dependent variable alcohol drinking was measured by two items that asked the respondents how many drinks per day and per week they had in the past month [62]. The two items were multiplied to calculate their weekly alcohol consumption.

The independent variable caregiving was the sum of five items, assessing the extent of caregiving burden by tallying care recipients [21, 63]. Respondents were asked if they currently provide care or make healthcare decisions for individuals with medical, behavioral, disability, or other conditions. Care recipients were classified into five categories: children, partners, parents, relatives, and friends, each coded as 0 for no and 1 for yes. Respondents were asked to select all applicable categories. As established in prior research [63], the number of care recipients is a significant indicator of caregiving burden. The greater the number of care recipients, the higher the caregiving burden. In this study, we summed all five care recipient categories to measure caregiving burden. This approach to caregiving construction has been validated in prior research [63]. The caregiving ranged from 0 (no responsibilities) to 5 (providing care for all five types of recipients).

The mediator psychological distress was the sum of four items (Cronbach’s α = 0.871) measuring the frequency with which the respondents experienced four symptoms of psychological distress in the past two weeks: (1) having little interest or pleasure in doing things; (2) feeling down, depressed, or hopeless; (3) feeling nervous, anxious, or on edge; and (4) not being able to stop or control worrying. This screening tool is adapted from the widely validated Patient Health Questionnaire for Depression and Anxiety (PHQ-4) [27, 64]. Each item used a four-point Likert scale (1 = not at all to 4 = nearly every day), which was linearly transformed to a 0 ~ 1 scale. Consequently, psychological distress ranged from 0 to 4, where 0 denoted no psychological distress and 4 indicated experiencing all four distress symptoms daily.

The first moderator, social media use for health, was the sum of four items that asked the respondents whether they had used social media for various types of health activities, such as participating in online forums, communicating on social media for health, or watching health videos on YouTube [65]. Each item was coded 0 or 1, where 0 indicated not conducting any kind of health activity on social media and 1 indicated performing an activity. Social media use for health ranged from 0 to 4, indicating 0 to 4 types of social media health use conducted by the respondents.

The second moderator, marital or romantic partnership, measured whether the respondents were living with romantic partners at the time of the survey, and was coded 0 for *no* and 1 for *yes* for analysis.

To reduce possible confounding effects, the demographic variables of *age*, *gender*, *education*, and *income* were included as control variables. See Table 1 for details.

**Table 1:** Descriptive statistics of the independent, dependent, mediating, moderating, and controlling variables (*n* = 3,865)

Dependent variable	
**Alcohol drinking** (Drinks/week, Mean ± SD)	3.41 ± 8.51
Independent variables	
**Caregiving** (Six levels 0 ~ 5, Mean ± SD)	0.18 ± 0.46
Mediation variable	
**Psychological distress** (Five levels 0 ~ 4, Mean ± SD)	0.67 ± 0.97
Moderating variables	
**Social media use for health** (Five levels 0 ~ 4, Mean ± SD)	1.25 ± 1.05
**Marital or romantic partnership** (n. %)	
with a partner	1,978 (51.2)
without a partner	1,743 (45.1)
Sociodemographic controls	
**Age** (years, Mean ± SD)	57.01 ± 17.00
**Gender** (n. %)	
Female	2,204 (57.0)
Male	1,561 (40.4)
**Education** ((n. %)	
Less than 8 years	80 (2.1)
8 through 11 years	193 (5.0)
12 years or completed high school	705 (18.2)
Post high school training other than college (vocational or technical)	264 (6.8)
Some college	817 (21.1)
College graduate	979 (25.3)
Post-graduate	684 (17.7)
**Income range** (Nine levels 1 ~ 9, Mean ± SD)	5.59 ± 2.26

### Data analysis

Data analysis was performed using SPSS (v26). We first conducted descriptive analyses for each of the variables. Second, Pearson correlation was performed to explore the associations. Furthermore, multivariate linear regression was conducted to test relations between variables in mediation and moderation models.

To reduce overreliance on significance tests and *p* values, we applied two effect size measures, *percentage coefficient (b*_*p*_*)* and *percent contribution (c*_*p*_*),* in this study to supplement familiar indicators such as *p* and *β* [66]. The percentage coefficient (*b*_*p*_) is a *b* coefficient when the dependent and independent variables are both linearly transformed into a percentage scale (*0* ~ *1*). Table 2 lists the descriptive statistics of each variable in natural and percentage scales. Percent contribution* (c*_*p*_*)* calculates the contribution of each path in the mediation model to the* X → Y* total effect [34–36]. Table 3 lists the calculation of *c*_*p*_ in detail.

**Table 2:** Variable Percentising

	Non-01 natural scales (*ns*)				Conceptual range		0 ~ 1 percentage scales (*ps*)			
	Min	Max	Mean	SD	Min	Max	Min	Max	Mean	SD
1. Alcohol drinking	0	210	3.41	8.51	0	100	0	2.1	0.03	0.09
2. Caregiving	0	5	0.18	0.46	0	5	0	1	0.04	0.09
3. Psychological distress	0	4	0.67	0.97	0	4	0	1	0.17	0.24
4. Social Media Use	0	4	1.25	1.05	0	4	0	1	0.31	0.26
5. Marital or romantic partnership	1	6	2.66	1.91	0	1	0	1	0.53	0.5
6. Gender-female	1	2	1.59	0.49	0	1	0	1	0.59	0.49
7. Age	18	104	57.01	17.00	0	100	0.18	1.04	0.57	0.17
8. Education	1	7	4.94	1.62	1	7	0	1	0.66	0.27
9. Income range	1	9	5.59	2.26	1	9	0	1	0.57	0.28

**Table 3:** Percent contribution to total X → Y effect (c_p_)

Indicator	Equation	Range	Eq
Percent contribution of total effect to total effect (*c*)	${c}_{p}\left(c\right)=\frac{{b}_{p}\left(c\right)}{|{b}_{p}\left(c\right)|}$	*c*_*p*_ (*c*) = 1 or *c*_*p*_ (*c*) = -1	1
Percent contribution of indirect effect (*ab*) to total effect (*c*)	${c}_{p}\left(ab\right)=\frac{{b}_{p}\left(ab\right)}{{|b}_{p}\left(c\right)|}$	-∞ < *c*_*p*_ (*ab*) < ∞ |*c*_*p*_ (*ab*)| ≤|*c*_*p*_ (*c*)|	2
Percent contribution of direct & remainder effect (*d*) to total effect (*c*)	${c}_{p}\left(d\right)=\frac{{b}_{p}\left(d\right)}{|{b}_{p}\left(c\right)|}$	-∞ < *c*_*p*_ (*d*) < ∞ |*c*_*p*_ (*d*)| ≤|*c*_*p*_ (*c*)|	3
Percent contribution of 1^st^-leg effect (*a*) to total effect (*c*)	${c}_{p}\left(a\right)=\frac{{|b}_{p}\left(a\right)|}{|{b}_{p}\left(a\right)|+{|b}_{p}\left(b\right)|}\times {c}_{p}\left(ab\right)$	-∞ < *c*_*p*_ (*a*) < ∞ |*c*_*p*_ (*a*)| ≤|*c*_*p*_ (*ab*)|	4
Percent contribution of 2^nd^-leg effect (*b*) to total effect (*c*)	${c}_{p}\left(b\right)=\frac{{|b}_{p}\left(b\right)|}{|{b}_{p}\left(a\right)|+{|b}_{p}\left(b\right)|} \times {c}_{p}\left(ab\right)$	-∞ < *c*_*p*_ (*b*) < ∞ |*c*_*p*_ (*b*)| ≤|*c*_*p*_ (*ab*)|	5

Before parenthesis. c_*p*_: percent contribution to total effect, *c*. *b*_*p*_: percentage coefficient

Within parenthesis. (*a*): first leg of the indirect path. (*b*): second leg of the indirect path

(*ab*): indirect path. (*d*): direct & remainder path. (*c*): total effect

## Results

### Preliminary analyses

Table 1 provides the demographic features of the respondents. The respondents were on average 57.01 years of age, with more women (57.0%) than men (40.4%), and 68.8% of the respondents reported an annual household income of above US$35,000. Among caregivers, approximately 87% reported providing care to one type of recipient and 13% reported providing compound care for different recipients. As shown in Table 4, most of our key variables were correlated. Caregiving was positively associated with psychological distress (*r* = 0.084, *p* < 0.01) and negatively associated with alcohol drinking (*r* = -0.038, *p* < 0.05), supporting the results of previous research [3, 4, 18, 33].

**Table 4:** Zero-order Pearson Correlations

Variables	1	2	3	4	5	6	7	8	9
1. Alcohol drinking	—	-.038*	.025	-.02	.015	-.124**	-.03	.043*	.088**
2. Caregiving		—	.084**	.121**	.114**	.078**	-.076**	.033*	.025
3. Psychological distress			—	.092**	-.106**	.093**	-.147**	-.090**	-.179**
4. Social Media Use				—	.088**	.125**	-.385**	.213**	.155**
5. Marital or romantic partnership					—	-.126**	-.080**	.115**	.420**
6. Gender-female						—	-.032	-.031	-.117**
7. Age							—	-.169**	-.184**
8. Education								—	.468**
9. Income range									—
*p < .05; **p < .01									

### Testing mediation

**Fig. 2:**
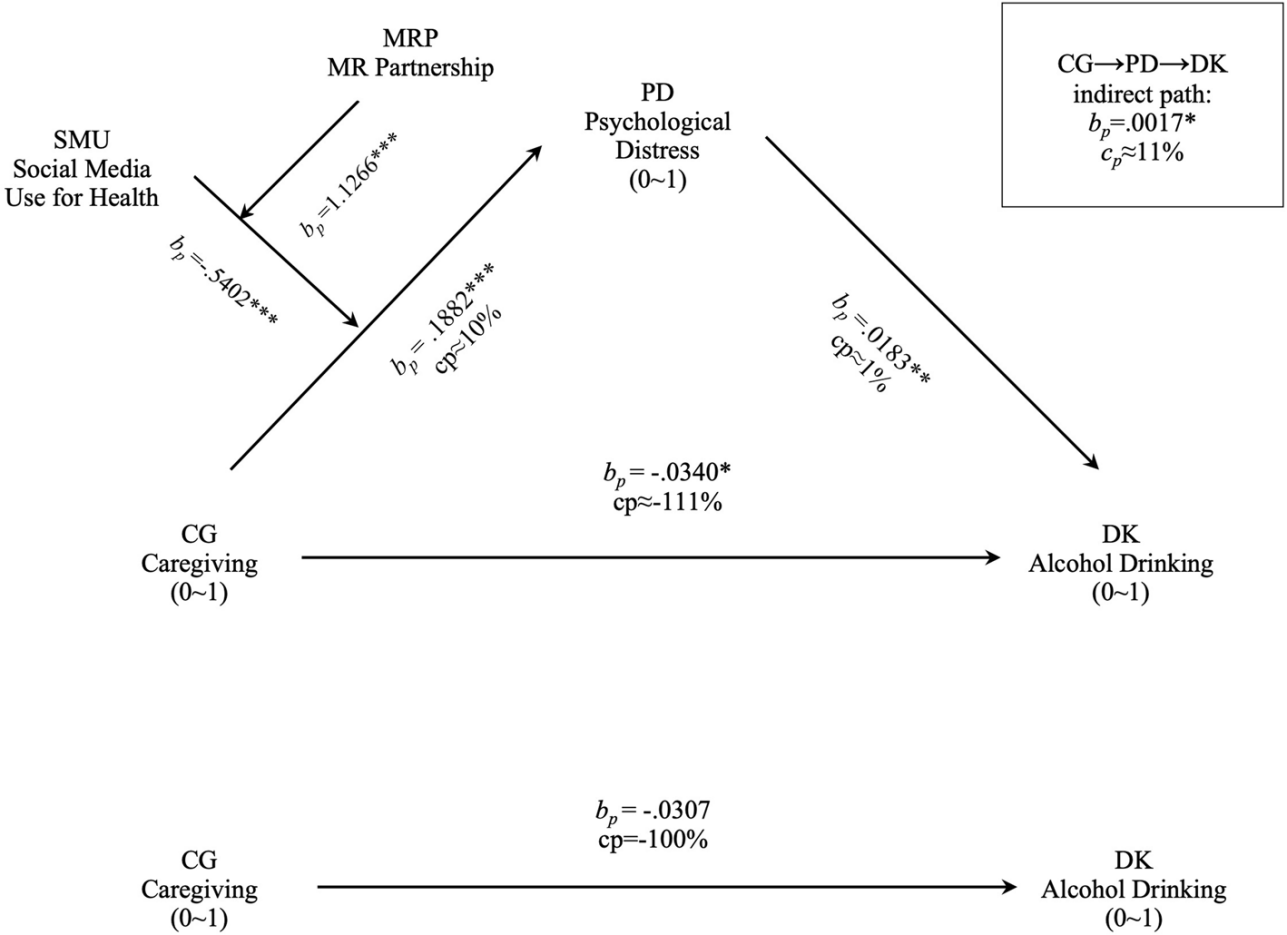
Effect of CG on DK mediated by PD and moderated by SMU and MRP. Note. * *p* < .05; ** *p* < .01; *** *p* < *.001*

Table 5 and Fig. 2 summarize the main findings, which we discuss below based on our hypotheses and research questions.

**Table 5:** Regression analyses of mediation and moderation effects

	Mediation Analysis		Moderation Analysis		Total Effect Analysis
Right: Equation ID	Equation I	Equation II	Equation III	Equation IV	Equation V
Right: Dependent Variable (DV)	Psychological distress	Alcohol Drinking	Psychological distress	Psychological distress	Alcohol Drinking
1. Intercept	.4031***	.0329***	.3716***	.3701***	.0403***
2. Female (FEM)	.0283 (.0578)***	-.0201 (-.1165)***	.0247 (.0503)**	.0218 (.0444)**	-.0196 (-.1135)***
3. Age (Age)	-.2563 (-.1801)***	-.0066 (-.0132)	-.2250 (-.1581)***	-.2234 (-.1570)***	-.0113 (-.0226)
4. Education (EDU)	-.0276 (-.0308)	.0017 (.0054)	-.0352 (-.0394)*	-.0406 (-.0455)*	.0012 (.0038)
5. Income Range (IR)	-.1654 (-.1931)***	.0240 (.0797)***	-.1682 (-.1963)***	-.1491 (-.1741)***	.0210 (.0697)***
6. Caregiving (CG)	.1882 (.0718)***	-.0340 (-.0369)*	.3987 (.1521)***	.7499 (.2861)***	-.0307 (-.0332)
7. Psychological distress (PD)		.0183 (.0518)**			
8. Social Media Use (SMU)			.0716 (.0776)***	.0906 (.0983)***	
9. SMU × CG			-.5402 (-.1059)***	-1.2543 (-.2460)***	
10. Marital or romantic partnership (MRP)				-.0122 (-.0254)	
11. MRP × CG				-.5266 (-.1720)**	
12. SMU × MRP				-.0298 (-.0310)	
13. MRP × SMU × CG				1.1266 (.1843)***	
Total *r*^*2*^	.075	.025	.081	.086	.022

^*^ *p* < .05; ** *p* < .01; *** *p* < .001

H1 predicts a positive link between caregiving and psychological distress (*a* path), and H2 predicts a positive linkage from psychological distress to alcohol drinking (*b* path). The results in Table 5 showed that the positive links between caregiving and psychological distress (*b*_*p*_ = 0.1882, *β* = 0.0718, *p* < 0.001, *cp* ≈ 10%) and between psychological distress and alcohol drinking (*b*_*p*_ = 0.0183, *β* = 0.0518, *p* < 0.01, *cp* ≈ 1%) were statistically acknowledged. Thus, H1 and H2 were both supported.

H3 predicts a positive indirect effect. As shown in Fig. 2, the positive indirect path from caregiving to alcohol drinking through psychological distress (a*b) was statistically acknowledged (*b*_*p*_ = 0.0017, Sobel test *p* < 0.05, bootstrap 95% CI [0.0001, 0.0045]), supporting H3. The indirect path (a*b) contributed a positive 11% of the total effect (*cp* ≈ 11%).

Q1 concerns the direction and the statistical test of the *d* path. As Table 5 (Eq. II) shows, the *d* path was negative and statistically acknowledged (*b*_*p*_ = -0.0340, *β* = -0.0369, *p* < 0.05, *cp* ≈ -111%). This path accounted for a significant negative contribution of -111% to the total effect.

Q2 concerns the total effect, i.e., the combined effect (*c* path). As shown in Table 5 (Eq. V), the *c* path was negative (*b*_*p*_ = -0.0307, *β* = -0.0332) but failed the statistical pretest (*p* = 0.062). As the total path showed the combined effect of the indirect path and the direct path, the conflicting directions led to a less negative but statistically inconclusive total effect.

### Testing moderation

H4 predicts a negative moderation effect of social media use for health on the caregiving to psychological distress path. Table 5 (Eq. III) shows a negative and statistically acknowledged moderation effect (*b*_*p*_ = -0.5402, *β* = -0.1059, *p* < 0.001), supporting H4.

H5 predicts a positive second-level moderation effect of marital or romantic partnership. As shown in Table 5 (Eq. IV), the interaction term (partnership × social media use × caregiving) was positive and statistically acknowledged (*b*_*p*_ = 1.1266, *β* = 0.1843, *p* < 0.001), supporting H5.

**Fig. 3:**
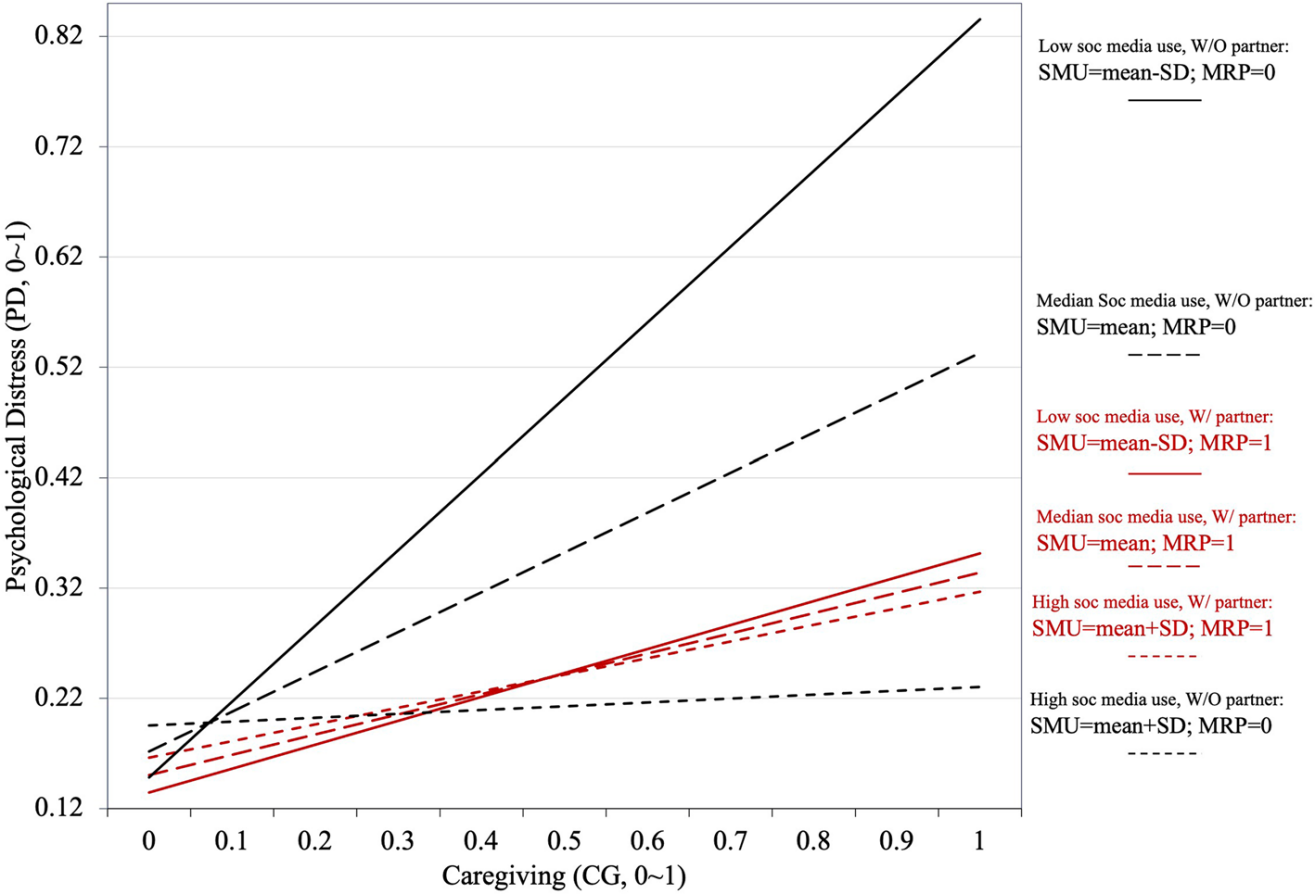
Moderated moderation effect of SMU and MRP on CG* → *PD effect (*a* path)

Figure 3 illustrates the moderated moderation effects (in percentage scales) on the first leg of mediation (*a* path). The effects of caregiving on psychological distress, although all positive, decreased when caregivers engaged in more social media health activities. Partnership showed the second-level moderation effect. For caregivers with partners, the moderation effect of social media use became weaker, while for those without partners this moderation effect was stronger. In the group without partners (black lines), for example, the lowest social media use (black solid) produced the steepest line, while the highest social media use (black dash) produced the flattest line among all groups. In the group with partners (red lines), the differences among the high, average, and low social media use sub-groups were smaller. Additionally, we observed that the caregivers with average social media use and with partners (red long dash) reported lower levels of psychological distress than those with average social media use but without partners (black long dash).

## Discussion

This research explored how different forms of communication and connectedness can help caregivers deal with their health issues. The relationships among caregiving, psychological distress, alcohol drinking, social media use for health, and partnership were tested using a moderated moderated mediation model, which is a model of mediation with two moderators that interact with each other.

We report an effect of mediation. Supporting findings of previous studies [3, 4, 8, 13, 29, 31], caregiving was positively associated with psychological distress, which was positively associated with alcohol drinking, producing a positive indirect association. As the caregiving workload increased (higher caregiving), the caregivers reported more symptoms of psychological distress (higher psychological distress). As psychological distress increased, they were more likely to use alcohol to relieve pressure. The identified indirect positive association supports the findings in previous studies that caregivers use drinking as an avoidance strategy to cope with psychological distress and stress [8, 31]. We also found a negative direct and remainder effect from caregiving to alcohol drinking, which partially supports previous findings of a negative association [4, 8, 33]. The different paths in the mediation model help to explain the seemingly contradictory findings concerning the association between caregiving and alcohol drinking identified in previous studies. This positive link highlights the levels of psychological distress caregivers can suffer from. Caregivers’ increased alcohol use can be regarded as a means of numbing themselves [18, 32, 39], which corresponds to the indirect path in our mediation model. However, when controlling for the effects of psychological distress, the direct and remainder association between caregiving and alcohol drinking was negative. Scholars who have identified a negative link have suggested that caregivers might use their personal resources when providing care to their families. The lack of energy and time, rather than psychological distress, is likely to account for the lack of social activity and their alcohol drinking [18, 26, 33].

Our findings reported a moderated moderation effect on the *a* path of the mediation model. Social media use for health was found to reduce psychological distress in caregivers, echoing previous findings [13]. As discussed, communication and connectedness effectively benefit caregivers’ well-being [11, 12, 41]. Social media, as the online form of communication and connectedness, provides caregivers with a time-convenient, cost-effective method of coping with psychological distress [13, 49, 50]. The communication on social media gathers caregivers together and generates emotional resonance based on similar experiences [13, 67]. Thus, caregivers who engage with social media for health tend to have more frequent connections and receive support, ultimately reducing psychological distress. The moderating effect of social media use can be identified. This study is among the first to examine the second moderation effect of partnership and the complex mechanism within online and offline communication and connectedness. Marital or romantic partnership, as the offline form of communication and connectedness, was found to positively moderate the moderation effect of social media use. For caregivers with partners, emotional affinity, intimacy, and togetherness, along with the physical forms of connectedness, such as caresses and hugs from partners, directly and effectively relieve their psychological distress [16, 17, 51, 52, 59]. Once their emotional demands are met, these caregivers may not seek other ways to release pressure. Thus, the moderation effect of social media use was reduced for caregivers with partners. Nevertheless, we want to emphasize that caregivers may fully make use of the combination of two measures, both the online and the offline forms, to obtain mental well-being. Prior research has indicated that diverse sources of communication and connectedness are more advantageous than single one [42, 43]. In our model, the groups who have both types of connectedness also reported fewer psychological distress symptoms (lower psychological distress) than many other groups.

We expanded on previous studies [22, 30] by investigating more detailed effects of caregiving. With the growing prevalence of compound caregivers [2], it's crucial to understand how the added responsibilities affect their mental well-being. Rather than a simple comparison between caregivers and non-caregivers or compound caregivers and non-compound caregivers, our findings revealed that as the number of care recipients increased, caregivers reported more symptoms of psychological distress.

## Implications

### Theoretical implications

In our mediation model, the indirect path revealed that caregivers increased their alcohol use in response to psychological distress [18, 32, 39], while the direct-and-remainder path indicated that caregivers drank less due to limited personal resources [18, 26, 33]. This mediation model provides a theoretical explanation that can inform the current divergent findings.

This study clarifies for the first time how different types of communication and connectedness affect caregivers’ mental health, and further reveals the underlying mechanisms of the interaction effects of online and offline forms of communication and connectedness. We extend the literature [13, 16, 17, 51, 52, 59, 67] by providing theoretical insights into various forms of communication and connectedness in terms of health. Online and offline forms of communication and connectedness have distinct and unique features. Social media for health offers caregivers various forms of support whenever and wherever they need it, while communication with partners directly provides caregivers with physical comfort and mental intimacy. The results suggest that, on average, the comfort and support from partners have a more powerful and direct influence on caregivers than social media use.

### Methodological implications

The effect size measures of *b*_*p*_ and *c*_*p*_ provide useful and novel information. In the mediation model, the mediation path contributed 11%, and the direct-and-remainder path contributed -111% to the negative total effect. The negative total effect from caregiving to alcohol drinking was mainly influenced by the direct-and-remainder path. Notably, the directions of the indirect and direct-and-remainder paths were reversed, leading to a competitive mediation [34, 68]. As *ab* + *d* = *c*, the positive *ab* and negative *d* effects resulted in a less negative but statistically inconclusive total effect, thus providing an explanation for some inconclusive findings in prior studies [4, 8]. Therefore, by exploring the effect size, we provide new methodological insights into the relationship between caregiving and alcohol drinking.

### Clinical implications

Our study also has potential practical implications. First, the increasingly aging population has led to an increase in the number of caregivers. We highlight concerns about the mental health of caregivers. By examining the mediation effect of psychological distress, we found that caregivers are likely to engage in risky health behaviors due to the mental burden of caregiving [4, 8]. Therefore, we suggest that public welfare organizations, health service professionals, and clinicians pay close attention to caregivers and their mental health.

Second, by examining the moderation effects of social media health use and partnership, we offer practical approaches that can benefit caregivers. Our findings revealed that 1) social media use had a greater effect on caregivers without partners; 2) on average, caregivers with partners reported fewer symptoms of psychological distress (lower psychological distress) than those without partners; and 3) those with a combination of high levels of social media use and partnership had fewer symptoms of psychological distress (lower psychological distress) than those in most of the other groups. Thus, we recommend that caregivers, particularly those with no partners, use social media for health-based communication. This represents a flexible, supportive, and convenient intervention that can mitigate psychological distress [13, 50]. Through health communities, caregivers can feel that they are supported and understood [13, 67]. Meanwhile, physical company and communication with partners are efficient a direct ways of reducing caregivers’ psychological distress [69]. As partnership further moderated the moderating effect of social media use, we emphasize its importance, although medical professionals may overlook this factor. Partnership and social media can together improve caregivers’ mental well-being. Thus, both online and offline forms of communication and connectedness should be provided to caregivers if possible.

We also note that caregivers are aging. Middle-aged or elderly caregivers may not be comfortable using social media to participate in health activities [70, 71]. Thus, the company and communication provided by partners can be an effective alternative. Additional supportive measures such as digital health education can also be promoted by health service professionals [72].

## Study limitations

We recognize several limitations in our study. First, the HINTS data were collected from a cross-sectional survey, and thus, we could not establish the causal relationships among caregiving, psychological distress, and alcohol drinking. In future research, experiments could be conducted to confirm these relationships. Second, HINTS is a nationally representative population‐based survey. Although aging is a global issue, the population structure, aging process, and caregiving system will vary from one region to another [23, 72]. New findings could therefore be obtained by examining various regions.

## Conclusion

This study demonstrated the mechanism through which caregiving affects alcohol drinking, as mediated by psychological distress and moderated by social media and partnership. This provides important theoretical and practical insights as the population ages and after the COVID-19 pandemic. Our model revealed a positive indirect path from caregiving to alcohol drinking through psychological distress and a negative direct and remainder path, thus providing a theoretical explanation for the previous mixed results concerning the association between caregiving and alcohol drinking. By analyzing the different effects of online and offline communication and connectedness, we found that social media use effectively improved caregivers’ mental health. Partnership further moderated the moderating effect of social media use. Based on our findings, we suggest that engaging in health activities through social media should be encouraged among caregivers. Physical company and communication are direct and powerful methods of improving caregivers’ mental status. Our findings suggest that the combination of online and offline communication and connectedness is more beneficial for caregivers’ health than a single approach.

## Data Availability

Data are available via http://hints.cancer.gov/.

## Abbreviations

CG: Caregiving
PD: Psychological distress
DK: Alcohol drinking
SMU: Social media use for health
MRP: Marital or romantic partnership
SD: Standard deviation
*b*_*p*_: Percentage coefficient
*c*_*p*_: Percent contribution
